# Editorial: *Legionella pneumophila*-transmission, pathogenesis, host-pathogen interaction, prevention and treatment

**DOI:** 10.3389/fmicb.2024.1364620

**Published:** 2024-01-31

**Authors:** Marta Palusińska-Szysz, Manuel Simões

**Affiliations:** ^1^Institute of Biological Sciences, Maria Curie-Skłodowska University, Lublin, Poland; ^2^LEPABE—Laboratory for Process Engineering, Environment, Biotechnology and Energy, Faculty of Engineering, University of Porto, Porto, Portugal; ^3^ALiCE—Associate Laboratory in Chemical Engineering, Faculty of Engineering, University of Porto, Porto, Portugal

**Keywords:** diagnosis, *Legionella* spp., legionellosis, prevention, sources, surveillance

*Legionella* species are rod-shaped and Gram-negative bacteria, considered opportunistic that act as an intracellular pathogen, and are the leading cause of legionellosis. *Legionella* spp., including *L. pneumophila*, are known to survive in complex ecosystems, including soil, natural and artificial water environments, being the presence of other microorganisms of relevance for their persistence. Some of the microorganisms associated with enhanced growth and survival of *Legionella* spp. include other bacteria, cyanobacteria, algae, and protozoa (Tison et al., 1980; McFeters, 1990). In particular, systems dense in protozoa and/or highly colonized by biofilms have a high risk of being contaminated by *L. pneumophila*. *Legionella* survival is potentially increased when in a biofilm (functional consortia of microorganisms adhered to a surface and to each other and/or embedded within extracellular polymeric substances, concentrated products of their metabolism, ions, and nutrients from the environment; Declerck, 2010).

There are more than 59 species and 70 serogroups of this bacterium (Springston and Yocavitch, 2017). Among the *Legionella* species, *L. pneumophila* is the prevalent cause of legionellosis. *L. pneumophila* has 15 identified serogroups, of which serogroup 1 is the main causative agent of legionellosis (Gonçalves et al., 2021b). Human infection by *L. pneumophila* can occur after the aspiration or inhalation of aerosols containing the bacteria. Upon infection, alveolar macrophages are invaded and used by *L. pneumophila* for replication. Virulence factors include flagella, fimbriae, types II and IV secretion systems, and iron-acquisition mechanisms. *L. pneumophila* outbreaks are increasing in occurrence regularity and are unavoidable, taking into account the current anthropogenic activities, the ineffective disinfection plans, and the source of the bacteria, as it is present in natural water and the soil. It is expected that *Legionella* outbreaks will continue increasing in numbers and severity, mostly because of the population aging in developed countries and the worldwide climate changes (Gonçalves et al., 2021a). The articles contained in this Research Topic provide a remarkable contribution to the diagnosis of *L. pneumophila* at the serogroup level. Additional insights on the genomic characterization and assessment of the pathogenic potential of *Legionella* spp. are provided.

In an outbreak scenario, the sampling process is critical for determining whether *L. pneumophila* is present and at what levels. Bacteria in the environment are typically in a viable but non-culturable state—VBNC (Colwell and Grimes, 2000). It means that even if the bacteria is present and viable they are not able to grow in the medium used. The recovery of VBNC *Legionella* is typically very difficult, requiring for most cases co-culturing with protozoa (Ramamurthy et al., 2014). The appearance of the VBNC state tends to increase when the microorganisms are exposed to stress conditions, such as disinfectants. In this Research Topic, Nisar et al. described a novel method to quantify VBNC *Legionella* from environmental water samples using a flow cytometry-cell sorting and qPCR assay. They demonstrated for the first time that flow cytometry-cell sorting in conjunction with qPCR is a rapid and direct method to quantify VBNC *Legionella* from environmental sources. Pascale et al. applied Fourier Transform Infrared Spectroscopy (FTIR) using the IR Biotyper^®^ system for the identification of *L. pneumophila* at the serogroup level. They found that the FTIR-based approach used is a powerful and easy-to-use approach to identify *L. pneumophila* serogroups and highlighted the relevance of the approach for outbreak investigations, allowing to trace the source of the infection and promptly adopt preventive and control strategies. In Tata et al., FTIR was used for *L. pneumophila* serogroup discrimination. The authors developed and validated a method, based on the coupling of FTIR and machine learning, for the automated serotyping of *L. pneumophila* serogroup 1, *L. pneumophila* serogroups 2–15 as well as their successful discrimination from *Legionella* non-pneumophila species. Girolamini et al. studied putative novel species associated with the *Legionella* genus. For that, they used a diversity of methods including cultoromics, MALDI-TOF MS, gene sequencing, and whole-genome sequencing analysis. In Svetlicic et al. four potential new species in the *Legionella* genus were identified, and functional annotations concerning virulence and antimicrobial resistance were performed on the sequenced genomes. Finally, Lehfeld et al. provided an analysis of community-acquired cases of legionellosis, particularly cases where infection was likely acquired at home. They highlighted the hypothesis that *Legionella* in oral biofilms or dental plaque may be a relevant cause of infections likely acquired at home.

While this Research Topic provides pioneer results on *Legionella* diagnosis, surveillance, and prevention, it is still clear that much remains to be understood on the evolutionary aspects of *Legionella* species, their mechanisms of transmission, accurate detection, and preventive and therapeutic strategies.

## Author contributions

MS: Conceptualization, Data curation, Formal analysis, Funding acquisition, Investigation, Methodology, Project administration, Resources, Software, Validation, Visualization, Writing – original draft, Writing – review & editing. MP-S: Conceptualization, Data curation, Formal analysis, Funding acquisition, Investigation, Methodology, Project administration, Resources, Software, Validation, Visualization, Writing – original draft.
